# Automated Mapping of Periglacial Landforms on Mars’ Utopia Planitia Using a Multi-Scale Texture-Enhanced U-Net

**DOI:** 10.3390/s26123653

**Published:** 2026-06-08

**Authors:** Xiaoyi Chang, Shuanggen Jin, Yanchao Zheng

**Affiliations:** 1 School of Surveying and Land Information Engineering, Henan Polytechnic University, Jiaozuo 454003, China; 212304010010@home.hpu.edu.cn (X.C.); zycjy0430@163.com (Y.Z.); 2 School of Artificial Intelligence, Anhui University, Hefei 230601, China

**Keywords:** periglacial landform, CTX, Utopia Planitia, MTU-Net

## Abstract

Martian periglacial landforms are among the clearest surface clues for investigating ground-ice occurrence, climate evolution, and potential habitability on Mars. Utopia Planitia contains abundant ice-related landforms and is therefore well suited to regional-scale mapping of periglacial features. However, most existing identifications still rely heavily on manual interpretation, which is time-consuming and difficult to keep consistent across large image mosaics. In this paper, using Context Camera (CTX) imagery, a dataset of four representative landform types in Utopia Planitia, namely flat-floored depressions, thermal contraction cracks, scalloped depressions, and brain terrain, was built. A Multi-scale Texture-enhanced U-Net (MTU-Net) was then developed as an automated and standardized mapping solution for semantic segmentation of these landforms. The model incorporates hierarchical attention and multi-scale texture enhancement modules, enabling recognition under complex backgrounds where fine-scale landforms such as thermal contraction cracks and brain terrain exhibit only weak textural details, alongside large scale variations. On the held-out test set, MTU-Net reaches a mean intersection over union (mIoU) of 89.55%, a mean F1-score of 94.71%, and a Kappa coefficient of 91.21%, outperforming the baseline U-Net under the same evaluation protocol. The resulting regional maps show marked spatial heterogeneity in the occurrence of the four landform types across Utopia Planitia. This study provides a methodological basis for automated periglacial landform mapping in Mars.

## 1. Introduction

The occurrence, migration, and degradation of water ice on the Martian surface and in the near-subsurface are key to understanding the Martian volatile cycle, orbital-scale climate variations, and the evolution of potential habitability [1,2]. Previous studies indicate that the mid-to-high latitudes of Mars preserve abundant geomorphological and subsurface evidence related to the stability of subsurface ice and climate oscillations. These records not only reflect the spatial distribution of near-subsurface ice but also provide important constraints for reconstructing recent climatic and environmental changes on Mars [3,4,5]. Recent grid-mapping efforts across the northern plains have integrated geomorphological mapping, Shallow Radar (SHARAD) sounding, and neutron spectrometer water-equivalent hydrogen data to characterize the distribution of ice-related landforms and their relationship with subsurface ice [6,7]. Therefore, identifying and characterizing ice-related landforms from remote sensing imagery is essential for understanding regional environmental evolution and the distribution of water ice resources on Mars.

Among various ice-related landforms, polygonal terrain, scalloped depressions, brain terrain, and their combinations are widely regarded as geomorphological responses to near-subsurface ice enrichment, thermal contraction cracking, sublimation degradation, and slope instability [8]. These processes indicate distinct near-surface environmental conditions: thermal contraction cracking requires ice-cemented ground and seasonal freeze–thaw cycles; sublimation degradation reflects the loss of excess ice under cold and dry atmospheric conditions; and slope instability is often associated with the melting or degradation of ground ice on steep slopes. All of these are characteristic of Martian periglacial environments and are directly linked to the formation and evolution of the specific landforms discussed below. Previous studies using High-Resolution Imaging Science Experiment (HiRISE), Mars Orbiter Laser Altimeter (MOLA), and regional mapping data have systematically investigated polygonal landforms, scalloped depressions, and brain terrain in Utopia Planitia and the mid-latitudes of the northern hemisphere, revealing their close relationships with ice-rich deposits, surface degradation, and climate change [9,10,11,12]. Thus, periglacial landforms are not only merely morphological features, but also serve as important geological records that constrain the history of near-subsurface ice activity on Mars. Systematic grid mapping in Utopia Planitia has identified three latitudinal assemblages of ice-related landforms, each correlated with ice-rich deposits of distinct ages and cryolithological characteristics [7].

Utopia Planitia, located in the northern lowlands of Mars, is a key region for studying water ice processes, sedimentary evolution, and paleoenvironmental changes [13,14,15]. This region is characterized by a wide variety of typical periglacial landforms and has long been recognized as preserving abundant information on near-subsurface ice activity [16]. Utopia Planitia is also one of the regions with the highest concentration of ice-related landforms in the mid-to-high latitudes of Mars, where scalloped depressions, thermal contraction polygons, brain terrain, and associated periglacial landform assemblages are widely developed. Previous studies have shown that scalloped depressions and small-scale polygonal landforms are widely developed in the western part of Utopia Planitia, between approximately 80° E–110° E and 35° N–50° N. Multiple types of periglacial landforms are also highly concentrated in the area of 80° E–105° E and 40° N–55° N. The formation of these landforms is generally closely related to processes such as sublimation degradation of near-surface ice-rich materials, thermal contraction cracking, and redistribution of surface materials [9,12]. Studies by Levy et al. further indicate that brain terrain on the surface of some concentric crater fill deposits in Utopia Planitia shows a clear connection to combined glacial and periglacial processes [11]. Meanwhile, thermal contraction crack polygons are also widely regarded as important morphological evidence of ice-cemented ground and cold-climate conditions [10].

In addition to surface morphological evidence, Utopia Planitia also holds significant research value for shallow subsurface ice and water. Radar detection by the Zhurong rover shows that beneath the shallow subsurface in the southern margin of Utopia Planitia, where the rover landed, there exists a multi-layered structure about 70 m thick [17]. These layered sequences are thought to preserve records of sedimentary and environmental evolution from the Late Hesperian to the Amazonian period in this region [15]. Concurrently, recent orbital and rover studies further suggest that the southern margin of Utopia Planitia may preserve various types of evidence related to paleo-coastlines, marine deposits, and ancient oceanic environments [18]. For these reasons, Utopia Planitia is not only a key region for studying near-surface volatile activity and periglacial landscape evolution on Mars but also an important target area for assessing the potential of water ice resources in the mid-latitudes of Mars [7]. Looking ahead, drone-based ground-penetrating radar (GPR) surveys could represent an important next step in identifying and mapping shallow subsurface ice on Mars, as illustrated by the FlyRadar targets proposed for future drone-based GPR investigations [19].

With the continuous accumulation of high-resolution remote sensing data from Mars, it has become possible to perform large-scale and detailed geomorphological identification using orbital images. The Context Camera onboard Mars Reconnaissance Orbiter (MRO), with a spatial resolution of about 6 m/pixel and wide swath coverage, has achieved near-global coverage of the Martian surface, providing a crucial data foundation for regional-scale identification and mapping of periglacial landforms [20]. However, existing research on periglacial landforms in Utopia Planitia and similar regions still largely relies on manual visual interpretation and expert experience. Although this method offers high reliability for detailed interpretation in small areas, it often suffers from low efficiency, strong subjectivity, and inconsistent results when applied to large-area CTX images. These challenges are exacerbated by diverse landform types, subtle textural differences, and complex backgrounds. This makes it difficult to meet the demands of automated mapping at the regional scale.

In recent years, encoder–decoder semantic segmentation networks represented by U-Net, along with improved models incorporating attention mechanisms, multi-scale feature extraction, and atrous convolution (also known as dilated convolution, which expands the receptive field by inserting holes between kernel elements without increasing parameters or losing resolution), have demonstrated strong capabilities in boundary recovery and multi-scale target recognition for remote sensing image segmentation [21,22]. These methods have also been gradually applied to automated Martian landform identification, including tasks such as impact crater segmentation, valley extraction, dune detection, landing site terrain classification, and weakly supervised geomorphological mapping, showing advantages over traditional manual feature extraction methods [23,24]. In addition, Zhao et al. [25] proposed a stepwise deep-feature transfer model for Martian landform mapping, achieving high-precision recognition with a small number of labeled samples through a two-stage transfer strategy. Notably, recent studies have begun to apply deep learning methods directly to automated mapping of Martian periglacial landforms. For brain terrain, Pearson et al. [26] used a convolutional neural network combined with a Fourier-domain classifier to automatically detect brain terrain across more than 52,000 HiRISE images, maintaining about 93% accuracy while saving about 95% of processing time, achieving the first Mars-wide automated mapping of brain terrain. For polygonal ground, Brito [27] employed U-Net and other deep learning models to classify and semantically segment polygons from HiRISE images, addressing binary classification, multi-class classification, and pixel-level segmentation. However, most of these studies have focused on only a single type of periglacial landform, such as brain terrain or polygonal ground. Simultaneous multi-category fine segmentation of the four typical periglacial landforms—namely flat-floored depressions, thermal contraction cracks, scalloped depressions, and brain terrain—remains very limited. Also, they have been reluctant to specifically address challenges such as weak textural details, large scale variations, and strong background interference [28,29,30,31].

Therefore, this study proposes a Multi-scale Texture-enhanced U-Net (MTU-Net) to map Martian periglacial landforms under complex backgrounds using CTX images. For identifying typical periglacial landforms on Mars’ Utopia Planitia, we construct a sample dataset containing flat-floored depressions, thermal contraction cracks, scalloped depressions, and brain terrain. Based on the U-Net framework, we introduce a multi-scale texture enhancement strategy to build the MTU-Net model, and improve the automatic segmentation accuracy and spatial distribution characterization of periglacial landforms under complex backgrounds.

## 2. Materials and Methods

### 2.1. Study Area

Utopia Planitia is located in the lowlands of the northern hemisphere of Mars and is one of the most representative giant low-lying basins on the Martian surface. According to the planetary nomenclature database of the International Astronomical Union (IAU) and the United States Geological Survey (USGS), the center of Utopia Planitia is approximately 49.67° N, 118.00° E, with a diameter of about 3200 km. Its extent covers latitudes from 14.83° N to 69.78° N and longitudes from 65° E to 160° E [32]. Utopia Planitia is situated northeast of Isidis Planitia, forming an important part of the northern lowlands of Mars. Using MOLA topographic data, Thomson and Head [33] characterized the Utopia basin as a circular, 1–3 km deep depression approximately 3200 km in diameter, confirming it as the largest easily recognizable impact structure in the northern hemisphere of Mars. The impact origin of the basin was first proposed by McGill [34], who identified a buried, topographically subdued basin covered by up to 1 km of sediments or lava flows. The global geological map of Mars from the USGS further shows that the overall elevation of the northern lowlands is significantly lower than that of the southern highlands, providing favorable conditions for sediment accumulation, ice-related processes, and surface modification [35]. In addition, recent studies have linked Utopia Planitia to a possible paleo-ocean in the northern lowlands of Mars, with evidence including shoreline-like features identified from MOLA data [36] and in situ observations of marine sedimentary rocks by the Zhurong rover [16].

**Figure 1:**
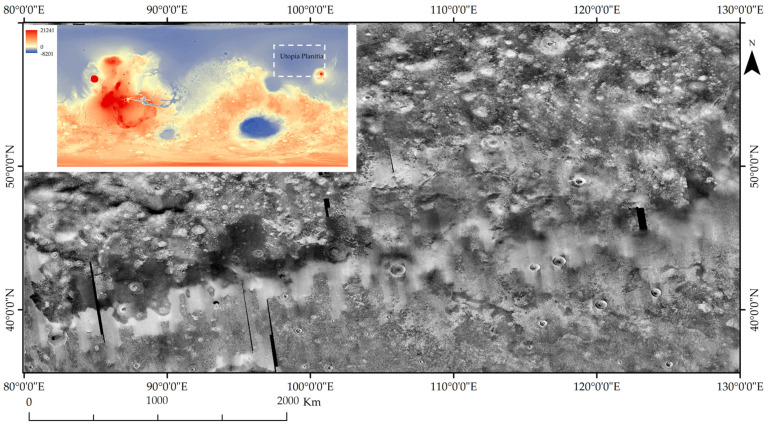
Study area map of the Utopia Plain (background image is CTX orbital image, and thumbnail is global MOLA DEM).

This study selects the mid-latitude portion of Utopia Planitia as the core study area, with a geographic range of 35° N–60° N and 80° E–130° E as shown in Figure 1. The area for dataset preparation is concentrated between 42° N–50° N and 68° E–86° E. This selection is based on three conditions. Multiple missions have found abundant near-surface water ice in this region. The Phoenix lander directly observed exposed water ice in the northern part [37]. SHARAD radar detected a thick ice-rich layer up to 170 m thick with 50–85% water ice content between 39° N and 49° N [11]. The Zhurong rover identified a 7 m thick ice layer at about 15 m depth in the southern margin [13]. Utopia Planitia also has the highest concentration and greatest diversity of periglacial landforms in the Martian mid-latitudes, including scalloped depressions, thermal contraction cracks, brain terrain, and flat-floored depressions [38]. In addition, the CTX global mosaic provides complete and high-quality image coverage over this region [38]. These conditions make Utopia Planitia an ideal area for developing and testing automated periglacial landform recognition methods.

### 2.2. Data Sources and Preprocessing

This study primarily uses CTX images from NASA’s MRO as the foundational data. Since 2006, CTX has been continuously acquiring high-resolution single-band images of the Martian surface. One of its design objectives is to provide regional context for other high-resolution instruments and to support regional-scale geomorphological surveys and mapping of the Martian surface [20]. Previous studies indicate that CTX images typically have a spatial resolution of about 5–6 m/pixel. To date, over 100,000 images have been acquired, achieving near-global coverage of the Martian surface. The latest global CTX mosaic, generated by Dickson et al. [39] using an information-preserving data processing workflow, covers approximately 99.5% of the Martian surface (88° S–88° N) at a uniform spatial resolution of 5 m/pixel. Compared to HiRISE data, which has higher spatial resolution but smaller coverage, CTX offers a better balance between regional coverage and spatial resolution, making it more suitable for regional-scale identification of periglacial landforms in Utopia Planitia.

This study uses the latest version (V01) of the global CTX mosaic of Mars, released by the Murray Lab at Caltech (https://murray-lab.caltech.edu/CTX/V01/tiles/ (accessed on 18 May 2026)), as the sole data source. This dataset is generated from raw images acquired by the Context Camera onboard the Mars Reconnaissance Orbiter, processed through professional radiometric calibration, geometric correction, and global mosaicking. It represents the highest-resolution global base image product of Mars currently available. The original GeoTIFF (Geographic Tagged Image File Format) image files covering the study area were obtained from the dataset website, with a uniform spatial resolution of 5 m/pixel, and the image quality meets the requirements for detailed identification of periglacial landforms. To balance local texture features and regional contextual information, the preprocessed CTX images were cropped into fixed-size sample patches with a certain overlap to prevent geomorphic features from being truncated at patch boundaries. Samples with significant stripe noise, severe shadow occlusion, or incomplete texture information were manually removed to ensure high-quality training data from the source. This step provides a stable data foundation for subsequent semantic segmentation model training and accuracy evaluation.

The CTX mosaic adopts a Plate Carrée equirectangular projection with equally spaced longitude and latitude grids. Under this projection, the actual ground distance per degree of longitude varies with latitude. At the equator, one degree of longitude corresponds to about 59 km; in the study area between 40° N and 55° N, it corresponds to approximately 45–38 km. The analysis is primarily based on coverage density statistics within 10° × 5° grid cells and on texture-based landform recognition. The local scale distortion introduced by the projection is nearly uniform within each cell, and therefore does not affect the relative trends of latitudinal zoning and longitudinal clustering. The scale bars in the figures indicate ground distances along the meridian (north–south direction). For east–west distances, the true scale is the displayed scale multiplied by cos (latitude), which ranges from ~0.77 at 40° N to ~0.57 at 55° N. The scale bars are provided for north–south reference only.

### 2.3. Dataset Construction

**Figure 2:**
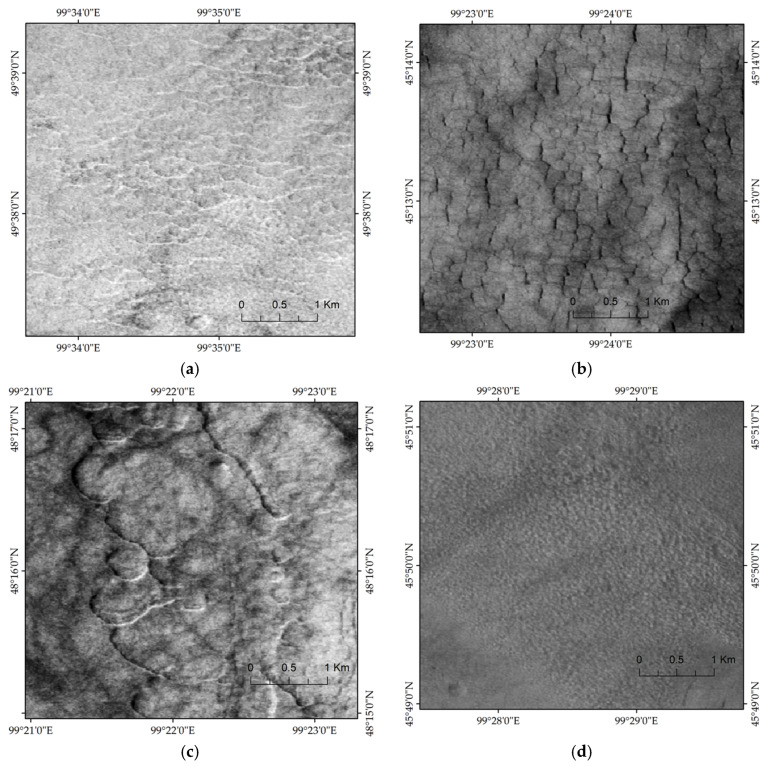
Image of four types of topographic features. All scale bars represent 1:25,000. (**a**) Flat-floored Depressions. (**b**) Thermal Contraction Cracks. (**c**) Scalloped Depressions. (**d**) Brain Terrain.

Dataset preparation was completed using the ArcGIS Pro 3.1.5. Based on the preprocessed CTX images, manual visual interpretation and vector annotation were conducted for four types of targets within the study area, combining the morphological characteristics of typical periglacial landforms in Utopia Planitia: flat-floored depressions, thermal contraction cracks, scalloped depressions, and brain terrain. As shown in Figure 2, flat-floored depressions appear as planar depressions with no marginal ridges and relatively flat floors. Scalloped depressions typically have arcuate or irregular scalloped boundaries, relatively flat interiors, and a certain asymmetry in slope aspect. Thermal contraction cracks mainly appear as reticulated or polygonal fracture systems. Brain terrain is characterized by banded, ridge-and-groove structures with a brain-like texture.

The preprocessed CTX images were cropped into fixed-size patches of 512 × 512 pixels with a 20% overlap, producing 1891 raw image-label pairs before filtering. Based on these annotations, this study paired image patches with their corresponding masks to construct a Martian periglacial landform sample dataset for semantic segmentation tasks. By removing blank patches where valid pixels accounted for less than 50%, a final set of 1585 image-label paired samples was obtained. The number of samples and image proportions for each category are shown in Table 1.

To improve annotation consistency, the initial manual labels were iteratively reviewed during dataset construction. Preliminary annotations were compared with intermediate model predictions, and regions with obvious discrepancies or ambiguous boundaries were re-examined and corrected according to the morphological characteristics of the target landforms. In addition, 15% of the samples were randomly selected for manual quality inspection. For this checked subset, the average pixel-level agreement IoU was 89.6% and the categorical agreement Kappa coefficient was 86.1%, suggesting that the finalized labels were sufficiently consistent for the subsequent experiments.

To enhance the model’s adaptability to variations in scale, texture direction, and local brightness, data augmentation operations such as rotation, flipping, random cropping, and intensity perturbation were applied to the training samples while preserving the basic morphology of the landforms. Subsequently, the samples were divided into training, validation, and test sets on a non-overlapping basis to prevent adjacent samples from entering different subsets, which could lead to overestimation of recognition accuracy.

To ensure fairness and comparability of model evaluations, all experiments followed a consistent data partitioning and evaluation protocol. The dataset was divided into training, validation, and test sets at a ratio of 7:1:2. The test set was used only for final model performance evaluation, effectively preventing data leakage from affecting the fairness of the experimental results and ensuring that different models were compared under consistent conditions, providing a reliable basis for subsequent performance evaluation. Because mixed pixels and fuzzy boundaries commonly exist between periglacial landforms and the surrounding background, further subdividing non-target areas would introduce significant subjective uncertainty and labeling noise. Therefore, all non-target geomorphic areas were uniformly classified as background, and their influence was minimized during model training and evaluation.

### 2.4. MTU-Net Model Architecture

**Figure 3:**
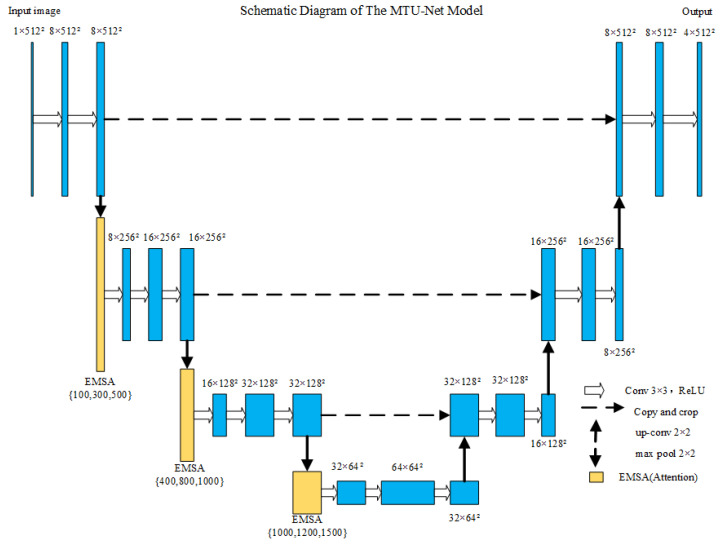
MTU-Net model architecture diagram. The model is based on the classic U-Net encoder–decoder structure, where the encoder includes three downsampling stages (left branch) and the decoder includes three upsampling stages (right branch). The numbers above each block (e.g., 8 × 512^2^) denote the number of channels and the spatial resolution (height × width) of the feature maps, respectively. The standard convolution block consists of a 3 × 3 convolution and a ReLU activation function, and the long dashed lines in the middle represent skip connections. The yellow blocks represent the EMSA module, which is placed before downsampling to capture multi-scale contextual information.

This study adopts U-Net [21] as the base architecture. To address the challenges in identifying Martian periglacial landforms, such as large scale variations among landforms, complex texture structures, imbalanced class distribution, and limited sample size, this study introduces targeted improvements to the model. A Multi-Scale Texture Enhanced U-Net (MTU-Net) model is constructed with its schematic architecture illustrated in Figure 3. This model uses U-Net as the backbone, retaining its symmetric encoder–decoder structure. Meanwhile, multi-scale texture feature extraction modules are embedded at different levels of the encoder, and the upsampling process in the decoder is optimized. These are combined with a specially designed data augmentation strategy and a compound loss function to enhance the model’s segmentation capability for the four types of periglacial landforms.

Compared with the original U-Net, the main improvements of MTU-Net are reflected in three aspects. First, an enhanced multi-scale texture module is introduced into the encoder to strengthen the representation of texture features across different spatial scales. Second, a hierarchical attention mechanism is incorporated from the middle to deep stages to improve the selection of informative regions and channels. Third, a compound loss function is designed to alleviate the effects of class imbalance and boundary ambiguity. Through these modifications, MTU-Net is better suited to the complex morphology and texture distribution of Martian periglacial landforms.

The encoder of MTU-Net consists of three downsampling blocks. The initial convolutional layer outputs 8 channels. The channel numbers progressively increase to 16, 32, and 64 after each downsampling block, while the bottleneck layer maintains 64 channels. The decoder symmetrically reduces the channel numbers back to 8 through upsampling blocks. The final output layer produces 5 class scores.

All main convolutional layers use a kernel size of 3 × 3 with padding of 1, including the residual blocks, the upsampling blocks, and the initial convolution. 1 × 1 convolutions are used for channel adjustment in shortcut connections, skip connections, and the texture module for channel compression and restoration. The Convolution Block Attention Module (CBAM) [40] spatial attention branch uses a 5 × 5 convolution, and the texture module predefines detection kernels of sizes 3 × 3 for Sobel and stripe patterns, and 5 × 5 for scallop and brain-terrain patterns.

Skip connections exist between the encoder and decoder. Each encoder stage returns a skip feature map via the residual block output, which is passed to the corresponding decoder layer. A 1 × 1 convolution adjusts the channel numbers of the skip features to match those of the upsampled output before concatenation.

For upsampling, bilinear interpolation followed by a standard convolution is used instead of transposed convolution. This avoids checkerboard artifacts, preserves spatial continuity, and ensures smoother feature maps. When the upsampled output and the skip feature have mismatched spatial sizes, interpolation or cropping is applied for alignment.

Batch normalization (BatchNorm2D) is applied after every convolutional layer, including residual blocks, the initial convolution, the texture module, feature fusion layers, and the final output layer. No other normalization methods, such as LayerNorm, are used.

The primary activation function is LeakyReLU with a negative slope of 0.1, used in most residual blocks and after downsampling and upsampling operations. ReLU is used only in the multilayer perceptron of the CBAM channel attention branch, and sigmoid is used for the attention gate output of CBAM.

### 2.5. Enhanced Multi-Scale Texture Module

The Enhanced Multi-scale Autocorrelation Texture Module (EMSA) is a core component of MTU-Net. Because different types of Martian periglacial landforms show significant differences in morphological scale, boundary structure, and textural features, traditional single-scale feature extraction methods struggle to capture both local detail expression and global structure perception simultaneously. To address this, this study designs a hierarchical scale allocation strategy. This allows the network to focus on texture scale ranges suitable for the current abstraction level at different depths, and employs a directional texture enhancement mechanism to improve the model’s ability to represent specific landform patterns.

Assume the input feature map of the EMSA module is $X\epsilon R^{B\times C\times H\times W}$, where *B*, *C*, *H* and *W* represent the batch size, number of channels, height, and width, respectively. First, a 1 × 1 convolution is used to compress the channels of the input feature to reduce subsequent computational complexity, expressed as:

(1) $$X_{r}={Conv}_{1\times 1}^{rea}(X)\epsilon R^{B\times C^{\prime }\times H\times W}$$

where $C^{\prime }=C/r$, and the compression ratio is *r* = 12.

Subsequently, the module selects the appropriate scale set based on the current network depth for subsequent operations. The shallow layer uses the small-scale set $S_{shallow}\epsilon \{100,\;300,\;500\}$ to capture the subtle undulations of the brain-like topography; the middle layer uses the medium-scale set $S_{middle}\epsilon \{400,\;800,\;1000\}$ to extract linear structures such as flat-floored depressions and thermal contraction cracks; while the deep layer uses the large-scale set $S_{deep}\epsilon \{1000,\;1200,\;1500\}$ to cover large-scale landforms such as scalloped depressions.

To ensure scale consistency across images with different resolutions, we employ an adaptive Gaussian kernel generation strategy. For each scale sϵS, calculating the relative scale $rel_{scale}\;=\;s\;/\;max(H,W)$, normalizing the ideal pixel scale s to the current image dimensions so that the subsequent filtering range can adaptively adjust according to the image resolution. We then dynamically determine the Gaussian kernel size based on Equation (2) [41].

(2) $$k_{s}=min(2\;\left[rel_{scale}\times min(H,W\right)]+1,\min\left(H,W\right)/2)$$

Here $k_{s}$ represents the side length of the Gaussian kernel. The relative scale $rel_{scale}\;=\;s\;/\;max(H,W)$ is normalized by the longer side of the feature map to ensure that the same scale s corresponds to a similar relative physical size across images of different resolutions. The upper bound of the Gaussian kernel size in Equation (2) is set to $\min\left(H,W\right)/2$ to guarantee that the kernel never exceeds the shorter side of the feature map, which is necessary for numerical feasibility. These two choices serve different purposes and are not contradictory. This expression uses rounding to ensure that the kernel size is odd. An odd-sized kernel has a well-defined center, which allows the convolution to be applied symmetrically around each pixel without shifting the feature map. At the same time, the kernel size is limited to $\min\left(H,W\right)/2$ or less, preventing the kernel from extending beyond the valid range of the feature map.

The standard deviation is calculated based on the product of the relative scale and the shorter side of the feature map, as shown in Equation (3) [41]:

(3) $$\sigma_{s}=max(rel\_scale\times \min\left(H,W\right),0.1)$$

The minimum value is set 0.1 to prevent the kernel function from degenerating due to an excessively small standard deviation. In practice, the Gaussian kernel is always computed within a finite window of size $k_{s}\times k_{s}$ as defined in Equation (2). Even if Equation (3) gives a large $\sigma_{s}$, the kernel is truncated to this window, and the weights outside are simply ignored. Therefore, an excessively large ${\;\sigma }_{s}$ does not cause numerical instability or unreasonable smoothing—it just means the kernel becomes nearly flat inside the window, which is acceptable for multi-scale texture extraction. Based on this, generate the Gaussian kernel [41] $G_{s}\epsilon R^{k_{s}\times k_{s}}$:

(4) $$G_{s}\left(i,j\right)=\exp\left(-\frac{i^{2}+j^{2}}{2\sigma_{s}^{2}}\right),\;i,j=-\left[k_{s}/2\right],\ldots ,[k_{s}/2]$$

Here, *i* and *j* represent the offsets of the elements within the kernel relative to the center, with values spanning the entire kernel region. This expression describes the probability density of a two-dimensional Gaussian distribution, such that elements closer to the center have greater weights, while those farther away have smaller weights. This effectively suppresses noise, preserves the main structure, and enables scale selectivity. After generation, the Gaussian kernel is normalized to ensure that the sum of all elements equals 1, thereby maintaining the overall brightness of the feature map before and after filtering.

Next, a grouped convolution is performed on the compressed feature map $X_{r}$ as shown in Equation (5):

(5) $$X_{blur,s}={Conv}_{group}\left(X_{r},G_{s}\right)\epsilon R^{B\times C^{\prime }\times H\times W}$$

Each group corresponds to a single input channel and uses the same Gaussian kernel $G_{s}$, independently to produce a blurred image. This is equivalent to applying Gaussian smoothing to each channel individually, thereby suppressing high-frequency noise while preserving low-frequency texture structures. The autocorrelation feature $A_{s}$ is then obtained using the Gaussian difference Equation (6) [42],

(6) $$A_{s}=X_{r}-X_{blur,s}\;\epsilon R^{B\times C^{\prime }\times H\times W}$$

The autocorrelation feature $A_{s}$ reflects the difference between the original feature map and its smoothed version $X_{blur,s}$, which is obtained via Gaussian filtering in Equation (5), effectively highlighting periodic patterns in local texture. When the texture exhibits a regular, repeating structure, Gaussian differentiation produces a significant response at the boundaries of the repeating patterns, while the response is weaker in the smoothed regions, thereby extracting texture information at a specific scale.

During the texture gradient extraction stage, to supplement the directional information missing from the autocorrelation features, the spatial gradient of $A_{s}$ is further computed. The Sobel operator $K_{x},K_{y}\in R^{3\times 3}$ is used to calculate the spatial gradient magnitude of $A_{s}$ and the gradient responses in the horizontal and vertical directions are extracted separately for group convolution. By applying the Sobel operator to each channel via group convolution, we obtain the gradient components $G_{x}={Conv}_{group}(A_{s},K_{x})$ and $G_{y}={Conv}_{group}(A_{s},K_{y})$. Subsequently, the gradient magnitude is calculated according to Equation (7) [42],

(7) $$G_{s}=\sqrt{G_{x}^{2}+G_{y}^{2}+\epsilon }\in \epsilon R^{B\times C^{\prime }\times H\times W}$$

Here $\epsilon =1\times 10^{-6}$ is a very small constant used to prevent numerical instability. The texture gradient feature $G_{s}$ enhances the edge response of the texture, effectively capturing regions of abrupt texture changes and directional variations; it complements the autocorrelation feature, and together they provide a more comprehensive description of the texture.

### 2.6. Hierarchical Attention Mechanism

MTU-Net adopts a progressive attention integration strategy that intelligently adjusts the intensity and complexity of the attention mechanism based on the network depth, balancing computational efficiency and feature quality [22,40]. The core concept of this strategy is that shallow features have high spatial resolution but low semantic abstraction; introducing excessive attention at this stage may interfere with the extraction of basic structural information. In contrast, deep features have reduced resolution but rich semantic information, requiring precise selection through attention mechanisms.

During the shallow feature extraction stage, specifically the initial convolutional layer and the first downsampling block, the model completely disables the attention mechanism. This stage primarily extracts basic features such as edges and corners, which are sensitive to spatial position and are already relatively sparse. The essence of the attention mechanism is to recalibrate feature responses by aggregating global information. Introducing such operations at the shallow stage may misclassify high-frequency noise as salient regions, thereby interfering with the purity of subsequent features. Therefore, no attention modules are introduced at this stage to ensure the stability of shallow features.

When processing mid-level features, specifically at the second downsampling block, the model begins to introduce a simplified Convolutional Block Attention Module (CBAM). This module consists of two sub-components: channel attention and spatial attention. Channel attention first applies global average pooling and global max pooling to the input feature map $F\in R^{B\times C\times H\times W}$, resulting in two 1×1×C channel descriptors. These are then passed through a shared two-layer perceptron to generate channel weights. Finally, the channel attention map $A_{c}$ is obtained after activation by the Sigmoid function, as shown in Equation (8) [40]:

(8) $$A_{c}=\sigma (MLP\left(AvgPool\left(F\right)\right)+MLP(MaxPool(F)))$$

Spatial attention takes the input feature map, computes the average and maximum across the channel dimension, generating two *H* × *W* spatial descriptors. These are concatenated along the channel dimension and passed through a 5 × 5 convolutional layer to produce the spatial weight map $A_{s}$, as shown in Equation (9) [40]:

(9) $$A_{s}=\sigma ({Conv}_{5\times 5}\left(\left[{AvgPool}_{c}\left(F\right);{MaxPool}_{c}\left(F\right)\right]\right))$$

A 5 × 5 convolutional kernel is used here instead of a smaller 3 × 3 kernel. Considering that the mid-level feature maps, namely the output of the second downsampling block, still have relatively high resolution, the 5 × 5 kernel ensures a sufficient receptive field while more effectively capturing spatial context information in local regions, avoiding the issue where a small kernel might be insufficient to accurately distinguish salient areas from the background. The compression ratio is set to 16 [40], balancing channel selection granularity, which refers to the level of detail in choosing which channels are kept after compression, and computational cost. The role of the attention mechanism at this stage is to preliminarily select feature channels and focus on salient regions, laying the foundation for subsequent refined processing.

At the deep-feature processing stage, specifically the third downsampling block and the bottleneck layer, the model fully employs an enhanced attention mechanism. The channel compression ratio remains at 16, and the spatial kernel size remains 5 × 5. Keeping these parameters consistent facilitates training stability and module reuse. Through this progressive configuration, the model maintains the purity of feature extraction in shallow layers while achieving precise feature selection in deep layers, effectively improving segmentation accuracy and training stability.

### 2.7. Evaluation Criteria

To comprehensively evaluate the performance of deep learning models in the task of identifying Martian periglacial landforms, this study adopts a multi-dimensional evaluation metric system. This system considers segmentation accuracy, class balance, and statistical significance, providing an objective reflection of the model’s overall performance from different perspectives.

For segmentation accuracy assessment, this study selects the following core metrics. Overall Accuracy [43] measures the proportion of correctly classified pixels among all pixels, reflecting the model’s general recognition capability. MIoU [43], as a core metric for semantic segmentation, calculates the intersection area over the union area between the predicted region and the ground truth region, which yields an overlap ratio ranging from 0% to 100%, being particularly sensitive to segmentation boundary quality. The Dice Coefficient [43] evaluates segmentation performance from the perspective of set similarity, making it especially suitable for handling class imbalance issues. The Kappa Coefficient [44] assesses the agreement between classification results and random classification, excluding the influence of chance agreement.

Considering the specific nature of the multi-class segmentation task, this study further refines the evaluation dimensions. Mean Precision and Mean Recall [43] evaluate model performance from the perspectives of exactness and completeness, respectively. Their harmonic means constitutes the Mean F1-Score [43], providing a comprehensive performance measure. Mean Accuracy (mAcc) [43] represents the average of class-wise accuracies, reflecting the model’s overall correct classification rate across different categories. Additionally, this study calculates IoU, Precision, and Recall independently for each landform category to perform an in-depth analysis of the model’s specific performance on different landform types.

## 3. Results and Analysis

Experiments were conducted based on the PaddlePaddle deep learning platform. The hardware configuration included a Tesla V100 GPU with 32 GB of memory, a 4-core CPU, and 32 GB of RAM, providing sufficient computational resources for large-scale model training. To evaluate the computational efficiency of the proposed MTU-Net, we compared the model size measured as number of parameters, training time, and inference speed in frames per second, Frames Per Second (FPS), with the baseline U-Net, DeepLabV3+ [45], and C2FNet [46]. Training settings were identical as well: input size 512 × 512 pixels, 800 epochs, batch size 24, Adam optimizer with an initial learning rate of $1\times e^{-5}$ using cosine decay with warmup. Inference FPS was measured with a batch size of 1, excluding post-processing steps such as argmax and resizing to the original image size. The results are summarized in Table 2.

MTU-Net introduces only a marginal increase in parameters, 0.23 million versus 0.22 million of the baseline U-Net, while achieving a 5.33 percentage point higher mIoU. Its training time, 11.08 h, and inference speed, 17.41 FPS, are comparable to those of U-Net. C2FNet has a large parameter count of 34.21 million but shows the shortest training time and highest FPS. This is because C2FNet adopts a simple coarse-to-fine two-stage architecture without complex attention or multi-scale texture modules, which allows faster computation per iteration despite its larger model size.

### 3.1. Model Performance Validation

To assess the contributions of the hierarchical attention mechanism and the multi-scale texture fusion module, we conducted ablation experiments using the original U-Net as the baseline. All experiments used the same training, validation, and test splits and the same evaluation protocol. Ablation experiments were configured with four settings: the baseline U-Net (Base), the baseline combined with the attention mechanism (+A), the baseline combined with the multi-scale texture module (+B), and the baseline combined with both modules. Table 3 summarizes the quantitative results, where ACIoU, BCIoU, CCIoU, and DCIoU denote the IoU values for flat-floored depressions, thermal contraction cracks, scalloped depressions, and brain terrain, respectively.

The baseline U-Net reached a mIoU of 84.22%, an F1-score of 90.86%, and a Kappa coefficient of 86.64% on the test set. At the class level, brain terrain (81.21%) and flat-floored depressions (83.48%) showed the lowest IoU values, which is likely related to their finer textures, subtle internal variation, and locally ambiguous boundaries. After introducing the hierarchical attention mechanism, the mIoU rose to 85.49%, the F1-score to 92.54%, and the Kappa coefficient to 88.27%. Improvements were observed across all four classes, with particularly clear gains for brain terrain, whose IoU increased from 81.21% to 83.79%, and scalloped depressions, whose IoU increased from 86.92% to 87.83%. These results suggest that attention helped the model suppress part of the background interference and place greater weight on informative regions.

When the multi-scale texture feature fusion module was introduced on its own, the improvement was larger, with the mIoU, F1-score, and Kappa coefficient increasing to 87.12%, 93.87%, and 90.33%. The IoU values of flat-floored depressions, thermal contraction cracks, and brain terrain rose to 86.32%, 87.39%, and 86.89%, respectively. This pattern suggests that explicit multi-scale texture modeling is particularly useful for landforms whose recognition depends strongly on local morphology and texture continuity. After both modules were combined, the full MTU-Net produced the best results, with a mIoU of 89.55%, an F1-score of 94.71%, and a Kappa coefficient of 91.21%. Relative to the baseline U-Net, the mIoU, F1-score, and Kappa coefficient improved by 5.33, 3.85, and 4.57 percentage points, respectively. The IoU values for flat-floored depressions, thermal contraction cracks, scalloped depressions, and brain terrain reached 89.02%, 88.39%, 91.88%, and 88.89%, respectively. For this task, texture modeling contributed more than attention alone, but the best performance was achieved when the two modules were used together.

**Figure 4:**
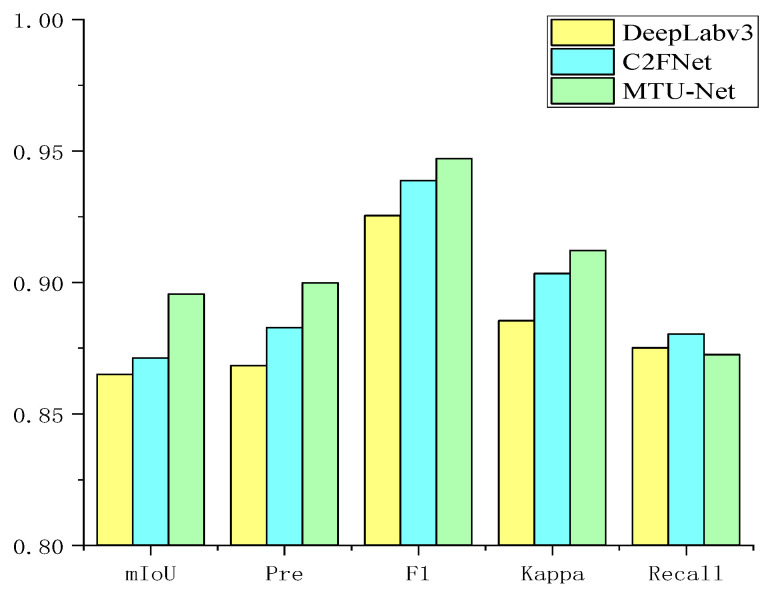
Quantitative evaluation of the three models on the test set.

To further evaluate the proposed MTU-Net, we compared it with two representative semantic segmentation models, DeepLabV3+ and C2FNet, on the same dataset. All models were evaluated under the same data split and evaluation protocol to ensure a fair comparison. Figure 4 shows the same pattern in the model comparison. MTU-Net yielded the highest mIoU, F1-score, and Kappa coefficient among the three models tested here. The advantage was particularly visible for landforms with irregular boundaries or dense internal texture, such as brain terrain and scalloped depressions, indicating that the combination of multi-scale texture enhancement and hierarchical attention was helpful for this dataset.

### 3.2. Verification of Identification Results

To further validate model performance qualitatively, four regions with well-developed and clearly textured typical periglacial landforms were selected for visual comparative analysis of the recognition results from the original CTX images, DeepLabV3+, C2FNet, and MTU-Net.

**Figure 5:**
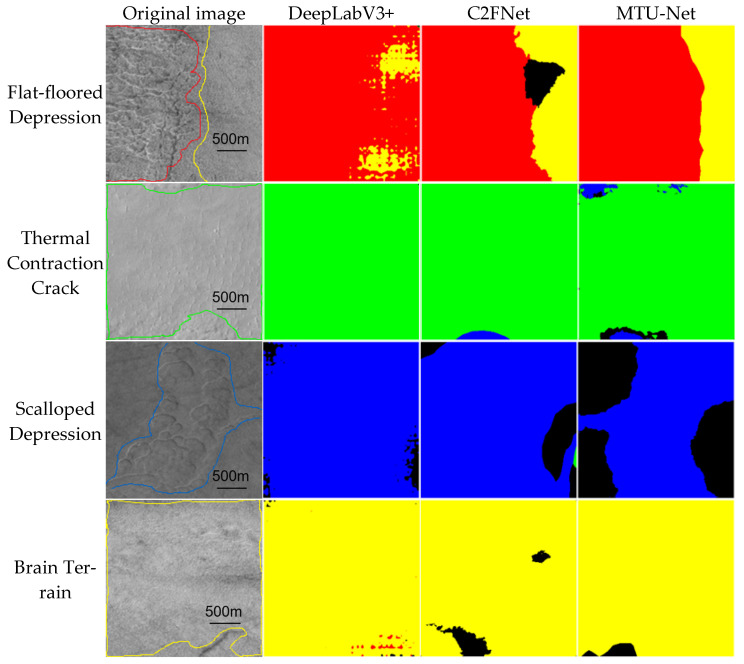
Comparison of typical visualizations for various terrain types (red indicates flat-floored depressions, green indicates thermal contraction cracks, blue indicates scalloped depressions, yellow indicates brain terrain, and black indicates background; the same below). CTX image ID: MurrayLab_V01_E088_N44_Mosaic (lower-left corner at 44° N, 84° E).

In Figure 5, the landforms are color-coded as follows: red for flat-floored depressions, green for thermal contraction cracks, blue for scalloped depressions, and yellow for brain terrain. Relative to DeepLabV3+ and C2FNet, MTU-Net recovers the outlines of the four landform types more completely in the selected examples. Flat-floored depressions are mapped with clearer boundaries, thermal contraction cracks retain more of their reticulate structure and small branches, scalloped depressions show better recovery of arcuate margins, and brain terrain is separated more cleanly from complex background textures. By contrast, DeepLabV3+ tends to miss weak textures and small targets, whereas C2FNet loses local detail or produces blurrier boundaries in some areas.

The visual examples are in line with the quantitative comparison. On this dataset, MTU-Net not only produced higher summary metrics but also preserved boundaries and textural detail more consistently in representative scenes.

### 3.3. Multi-Source Verification

#### 3.3.1. Superposition Analysis with the USGS Geological Map

To verify the geological plausibility of the MTU-Net recognition results, the spatial distribution maps of the four types of periglacial landforms were superimposed with the USGS global geological map of Mars, and the area proportions of each landform type within different geological units were calculated, as presented in Table 4. If the model recognition results are consistent with the geological background—that is, specific geological units exhibit systematic preferences for certain landform types—then the geological reliability of the model predictions can be corroborated.

Fa et al. [47] systematically analyzed the shallow subsurface structure and material composition of six major geological units in Utopia Planitia using SHARAD radar data. Soare et al. [48] further indicated that the dark volcanic terrains in this region are composed of fine-grained volcanic materials, which favor the formation of ice-wedge polygons and near-surface excess ice. Based on these studies, the surface materials of the AHv unit (Amazonian and Hesperian volcanic unit) and Av unit (Amazonian volcanic unit) are predominantly dense volcanic lava, and the topographic evolution is mainly controlled by volcanic activity. Such dense caprocks are unfavorable for the detailed modification by subsequent periglacial processes. Consistent with this geological expectation, the total area proportion of periglacial landforms identified by the model in these two units is relatively low, and the landform types are singular, with brain terrain being absolutely dominant. In the AHv unit, brain terrain accounts for 92.83% of the identified periglacial landforms, while in the Av unit, brain terrain accounts for 66.91%, with the remaining three landform types together comprising less than 35%. This result is highly consistent with the understanding that volcanic units are not conducive to the widespread development of periglacial landforms.

For the lHl unit (Late Hesperian lowland), eAb unit (Early Amazonian basin), mAl unit (Middle Amazonian lowland), and AHi unit (Amazonian and Hesperian impact unit), Fa et al. [47] noted that their surface materials are mainly composed of fine-grained sediments of the Vastitas Borealis Formation (VBF). Xiao et al. [16] further confirmed through observations from the Tianwen-1 rover that the VBF fine-grained sediments exhibit characteristics of marine sedimentary rocks, possibly originating from ancient marine evaporite residues or flood alluvial deposits. Fine-grained sediments provide an ideal material basis for periglacial processes and are expected to host diverse and high-density periglacial landforms. The model recognition results fully align with this expectation. In the lHl unit, flat-floored depressions account for the highest proportion at 48.80%, followed by brain terrain, consistent with the understanding that fine-grained sediments favor the development of linear fractures and reticulated textures. In the eAb and mAl units, the four landform types are relatively evenly distributed, with thermal contraction cracks accounting for a lower proportion, reflecting the superimposed characteristics of multiple phases of periglacial activity, which is consistent with the geological evolution expected for basin sedimentary units. The AHi unit features the most rugged topography, and the model recognition results are dominated by scalloped depressions and brain terrain, with flat-floored depressions accounting for only 4.98%. This aligns with the expectation that rough terrain inhibits the development of fine linear fractures but facilitates the formation of large depressions and complex textures.

Furthermore, Soare et al. [38] indicated the presence of an ice-rich, loess-like fine-grained unit in the mid-latitudes of Utopia Planitia, which serves as the parent material layer for the development of periglacial landforms such as scalloped depressions and polygonal terrain. The model recognition results show that even within the AHv unit, which is dominated by volcanic lava, brain terrain still accounts for as much as 92.83% of the identified periglacial landforms, while the sum of the other three landform types is less than 8%. This suggests that although the AHv unit is primarily composed of volcanic lava, local remnants or subsequent coverage of ice-rich fine-grained materials may be preserved, allowing the selective development of brain terrain. This finding is consistent with the view proposed by Soare et al. [49] that periglacial landforms are not strictly confined by geological units but are primarily controlled by the distribution of shallow subsurface water ice, thereby further validating the geological sensitivity of the model recognition results.

In summary, the area proportions of periglacial landforms identified by the model within each geological unit are highly consistent with geological expectations derived from the literature. In volcanic lava units AHi and AHv, periglacial landforms cover only 7.8% and 0.05% of the unit area respectively, with thermal contraction cracks being the dominant type. In fine-grained sedimentary units eAb and mAl, periglacial landforms cover 31.6% and 22.0% of the unit area, respectively, exhibiting a diverse assemblage including flat-floored depressions, thermal contraction cracks, scalloped depressions, and brain terrain. In rugged impact units lHl and Av, periglacial landforms cover 2.7% and 5.6%, respectively, with flat-floored depressions as the predominant type, accounting for more than 60% of periglacial features in these units.

#### 3.3.2. Verification by HiRISE Images

**Figure 6:**
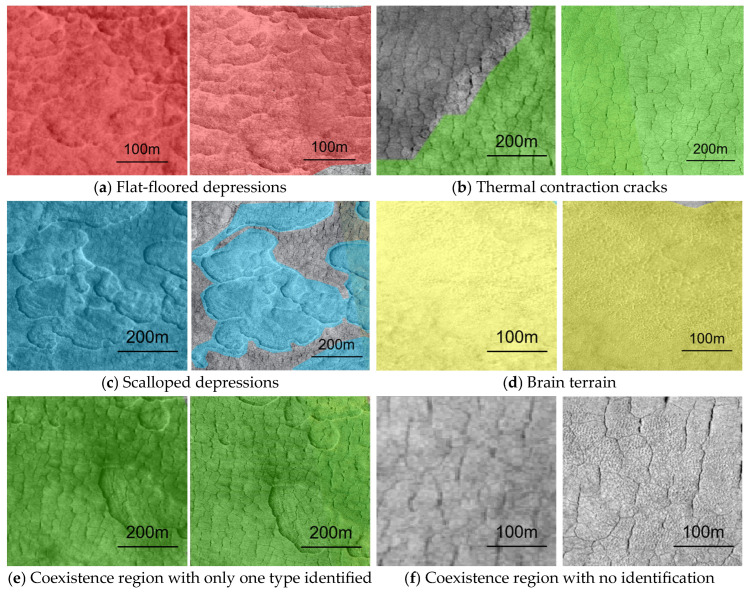
Comparison and verification of typical regions. Left panels are CTX images overlaid with model prediction results. Right panels: (**a**–**d**) are HiRISE images with manually annotated ground truth for the dominant landform types; (**e**,**f**) are HiRISE images overlaid with the same model prediction results as shown in the left panels to confirm the presence of landforms in these areas. The right panels show cropped subregions of the original HiRISE images. Their full image IDs and center coordinates are as follows: (**a**) ESP_084843_2260_MRGB, 45°51′0″ N, 86°31′1.2″ E; (**b**) ESP_079239_2245_MRGB, 44°3′7.2″ N, 87°45′25.2″ E; (**c**) ESP_078316_2260_MRGB, 45°35′24″ N, 87°11′45.6″ E; (**d**) ESP_088166_2265_MRGB, 46°19′48″ N, 85°18′39.6″ E; (**e**) ESP_087955_2260_MRGB, 45°33′57.6″ N, 86°21′21.6″ E; (**f**) ESP_080782_2245_MIRB, 44°10′44.4″ N, 86°26′24″ E.

The resolution of CTX images limits the ability to represent fine-scale geomorphic details. To evaluate the recognition limits of the model at the CTX scale, five HiRISE images with a resolution of approximately 0.3 m/pixel, covering areas of the four typical periglacial landforms near 45° N and 83° E, were selected for visual comparison and verification, as shown in Figure 6.

The comparison results indicate that in regions dominated by flat-floored depressions, the model predictions match the true morphology revealed by HiRISE images well, with accurate boundary delineation and no obvious omissions or misclassifications. In areas where brain terrain is concentrated, the model accurately captures its macroscopic outlines, and the recognized extent is generally consistent with the ridge-and-groove textures observed in the HiRISE images. To quantitatively assess the model’s performance at the CTX scale, we manually annotated landform boundaries on the HiRISE subregions shown in Figure 6a–d and calculated the IoU against the CTX-based predictions. For flat-floored depressions Figure 6a, the IoU reached 97.00%, indicating excellent agreement. For brain terrain Figure 6d, the IoU was 95.46%, also demonstrating high accuracy. However, for thermal contraction cracks Figure 6b and scalloped depressions Figure 6c, the IoU values were 47.46% and 63.98%, respectively, reflecting the model’s difficulty in resolving fine linear features and low-contrast boundaries from CTX imagery. These quantitative results are consistent with the visual comparison in Figure 6e,f and highlight the model’s strengths in homogeneous terrains and its limitations in complex mixed scenes.

However, in complex scenes where multiple landform types coexist, the model performance exhibits significant limitations. Where thermal contraction cracks are tightly interwoven with brain terrain, the model shows clear omissions in these regions and fails to effectively separate the two landform types. Where scalloped depressions coexist with thermal contraction cracks in the same area, the model displays a recognition bias toward scalloped depressions, identifying only the scalloped depressions while completely missing the coexisting thermal contraction cracks. In scenes where thermal contraction cracks and scalloped depressions are intimately coexisting throughout the entire image with comparable proportions, the model strongly tends to recognize thermal contraction cracks, leaving the scalloped depressions entirely undetected. Furthermore, in regions where thermal contraction cracks and brain terrain coexist across the whole image, the low grayscale contrast and blurred textures of both landform types in the CTX images cause the model to fail completely, misclassifying the entire area as background.

The above comparison and verification reveal the core bottlenecks of MTU-Net under the CTX data source: landforms with complex textures, such as brain terrain, and fine linear features, such as low-contrast thermal contraction cracks, are prone to omission or confusion in areas of coexisting landforms. This corroborates the analysis of misclassification cases presented in Section 3.1. Nevertheless, the model’s recognition results in macroscopic, single-landform-dominated areas are of reference value and are sufficient to support the analysis of large-scale spatial distribution patterns in the study area.

The superposition analysis with the USGS geological map shows that periglacial landform proportions within each geological unit are highly consistent with geological expectations. In volcanic units AHv and Av, periglacial landforms cover only a small fraction of the unit area and are dominated by brain terrain. In fine-grained sedimentary units lHl, eAb, mAl, and AHi, which are associated with the Vastitas Borealis Formation, periglacial landforms cover a substantial proportion of the unit area and exhibit diverse assemblages including flat-floored depressions, thermal contraction cracks, scalloped depressions, and brain terrain. The comparison with HiRISE images reveals model limitations under CTX resolution constraints, specifically omission and category bias in complex regions where multiple landform types coexist. Nevertheless, macro-scale statistics based on a 10° × 5° grid effectively buffer local misclassifications, and the overall pattern of latitudinal zonation and longitudinal compartmentalization remains robust.

### 3.4. Spatial Distribution of Periglacial Landforms

Based on the MTU-Net predictions for CTX images in the mid-latitude portion of Utopia Planitia, we mapped the regional distribution of four typical periglacial landforms: flat-floored depressions, thermal contraction cracks, scalloped depressions, and brain terrain. A grid of 10° longitude by 5° latitude was then used to calculate the coverage density of each landform type and to summarize their regional spatial heterogeneity. Overall, the mapped landforms show a distinctly non-uniform distribution, with both latitudinal zoning and longitudinal clustering.

**Figure 7:**
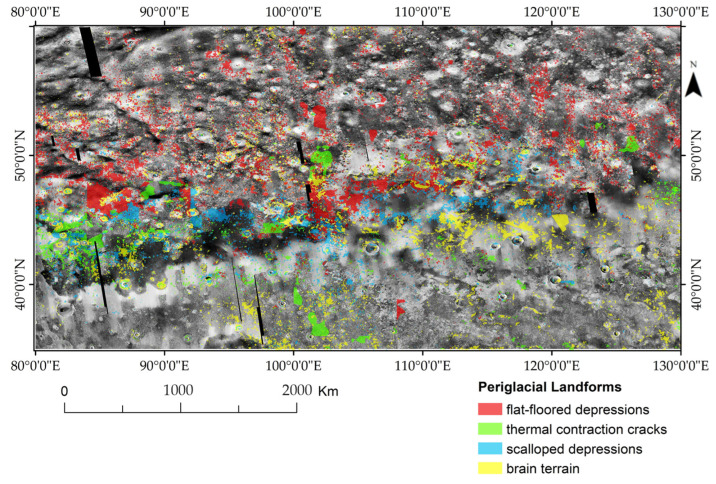
Spatial distribution of four-type periglacial landforms on the Utopia Planitia.

As shown in Figure 7, the four landform types exhibit both spatial differences and partial spatial associations. Thermal contraction cracks are mainly concentrated in the western to north-central part of the study area, where they commonly form continuous reticulate or banded patterns. Brain terrain is more concentrated in the eastern part of the study area and commonly occurs in contiguous patches with fine and continuous textures. Flat-floored depressions have the broadest distribution and are widely present among different geomorphic units, whereas scalloped depressions—arcuate depressions with flat floors and asymmetric slopes formed by sublimation degradation—are relatively sparse and tend to occur in local clusters in the mid-western part of the study area. At the regional scale, these results indicate clear heterogeneity in the spatial occurrence of the four landform types.

**Figure 8:**
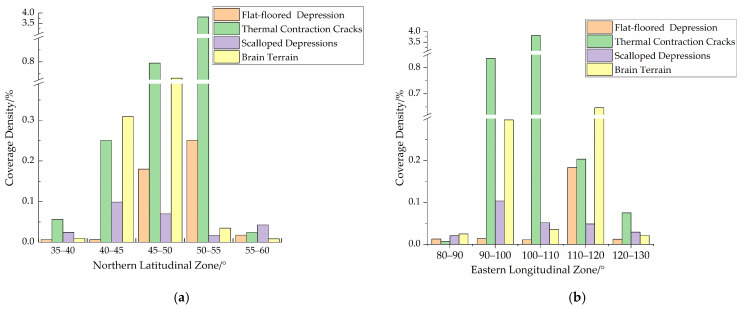
Statistical maps showing the coverage density of four types of periglacial landforms. (**a**) shows statistics by latitude band and (**b**) shows statistics by longitude band.

To quantitatively analyze the spatial differentiation characteristics of the four landform types, the coverage density of each landform type in different latitudinal and longitudinal zones was calculated, with results shown in Figure 8.

In the latitudinal direction, thermal contraction cracks and flat-floored depressions show their highest densities mainly between 50° and 55° N, whereas scalloped depressions and brain terrain are more concentrated between 40° and 50° N.

In the longitudinal direction, the four landform types also show clear local differences. Thermal contraction cracks are most concentrated between 100° and 110° E, brain terrain between 110° and 120° E, and scalloped depressions between 90° and 100° E. Flat-floored depressions are the most widespread landform type, but they also show a local peak near 110° to 120° E. This pattern suggests that latitude alone does not explain the mapped distribution and that local geomorphic and geological settings probably also influence the landform assemblages.

To summarize the mapped distribution patterns and their possible geomorphological interpretations, Table 5 lists the main concentration zones and the corresponding interpretations for each landform type.

### 3.5. Typical Landforms and Spatial Relationships

To further demonstrate the consistency between the model recognition results and typical landform morphologies, four areas with well-developed and clearly textured typical periglacial landforms were selected for detailed analysis, with results shown in Figure 8.

**Figure 9:**
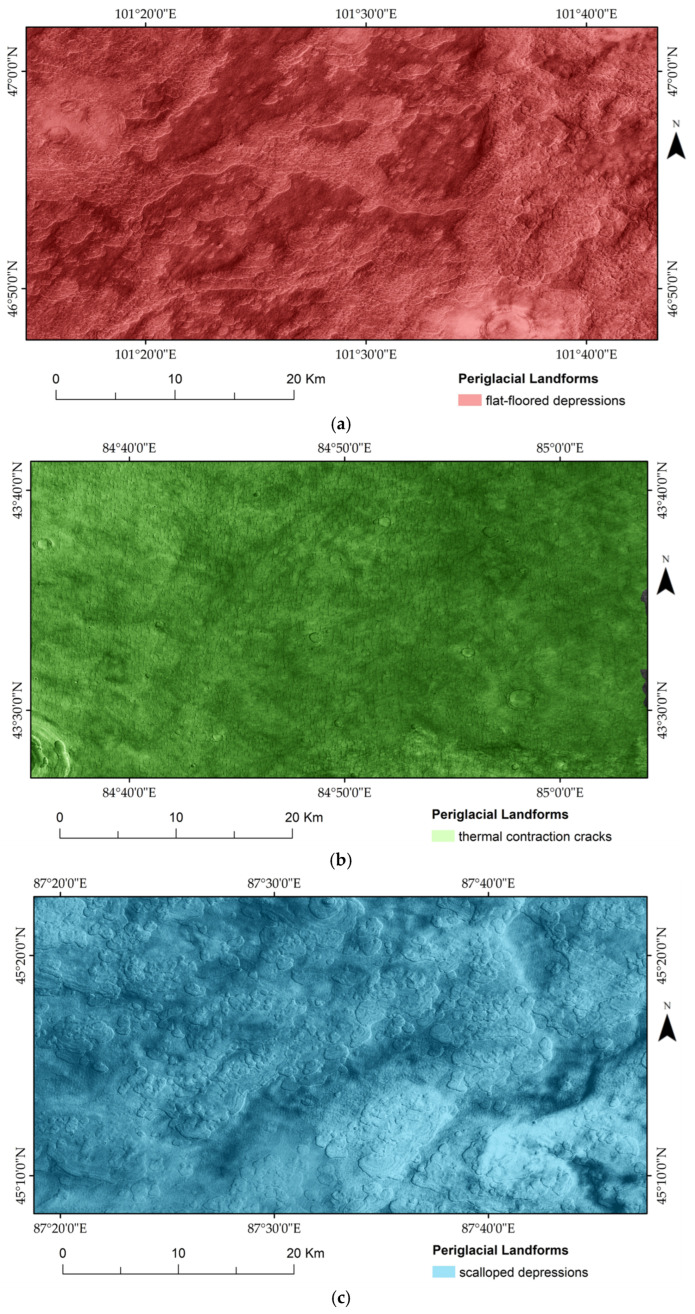

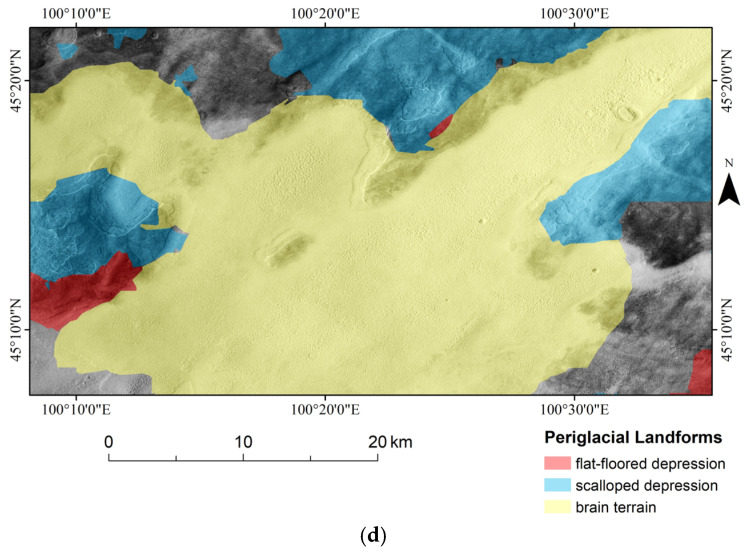
Detailed view of typical periglacial landforms and their spatial patterns, showing (**a**) a flat-floored depression, (**b**) thermal contraction cracks, (**c**) a scalloped depression, and (**d**) brain terrain.

Based on these representative areas shown in Figure 9, the flat-floored depressions generally appear as shallow depressions with relatively clear boundaries and flat floors, often scattered among other geomorphic units. Thermal contraction cracks appear as regular or semi-regular linear grid textures and are among the most typical periglacial landforms in the study area. Scalloped depressions commonly show arcuate or irregular scalloped margins and relatively flat interiors, whereas brain terrain displays continuous ridge-and-groove textures with strong internal continuity. In terms of spatial association, flat-floored depressions commonly occur adjacent to thermal contraction cracks and brain terrain, while scalloped depressions and brain terrain are locally associated in some areas. Combined with the regional maps, these examples suggest that the periglacial landforms in Utopia Planitia are spatially heterogeneous and may reflect the combined influence of regional latitudinal gradients and local geomorphic settings.

**Figure 10:**
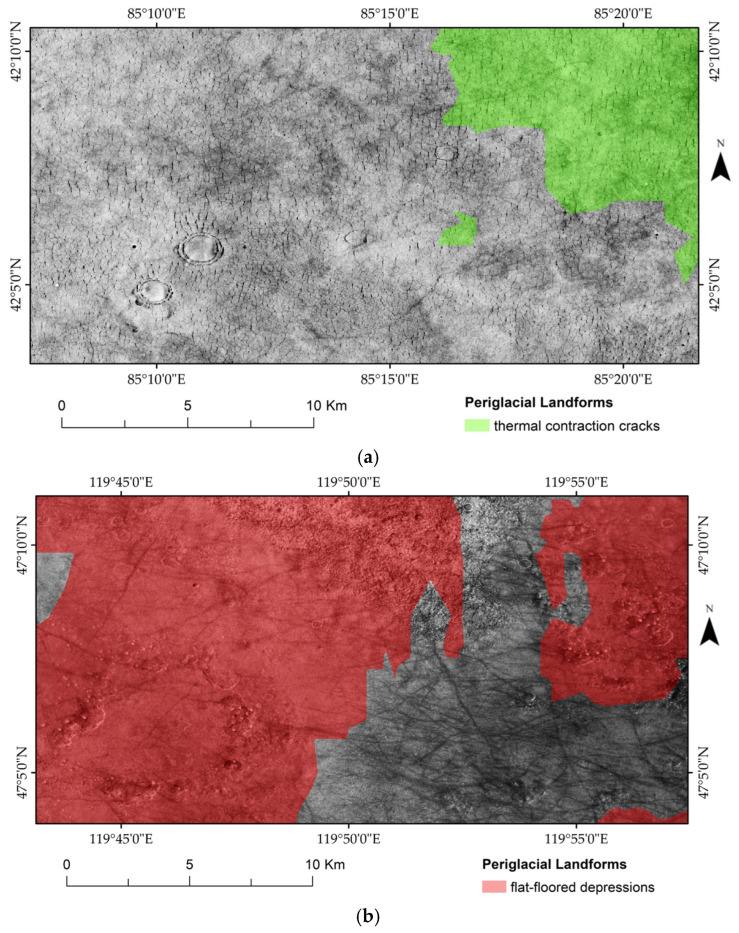
Examples of recognition uncertainty, where (**a**) shows a missed classification and (**b**) shows a misclassification.

Typical examples of missed and misclassified cases in model recognition are illustrated in Figure 10. As shown in Figure 10a, thermal contraction cracks in the western part of the study area exhibit narrow linear patterns. In regions with gentle topography and uniform illumination, the textural contrast between these cracks and the background grayscale is weak, making it difficult for the model to detect many scattered cracks, which leads to an underestimation of thermal contraction crack density in that area. Furthermore, CTX is a single-band grayscale image, and landform recognition relies entirely on textural features. Deep learning models are highly sensitive to texture patterns—this is an advantage for extracting complex landforms, but it also becomes a source of misclassification when different landforms share similar textures. For example, as illustrated in Figure 10b, aeolian streaks in some areas closely resemble the texture of flat-floored depressions, and crater rims can appear similar to scalloped depressions.

Consequently, the model misclassifies extensive background textures as target landforms, artificially increasing the coverage density of flat-floored depressions and potentially biasing the distribution patterns. The model still has certain limitations in distinguishing landforms with similar textures, such as confusion between flat-floored depressions and thermal contraction cracks, or between brain terrain and some background textures, which may affect local statistical values.

## 4. Discussion

The spatial distribution of periglacial landforms in Utopia Planitia exhibits a distinct pattern of latitudinal zonation. Thermal contraction cracks and flat-floored depressions are concentrated at higher latitudes, indicating a strong spatial association with the current near-surface water ice stability zone. This observation is highly consistent with the thermal–physical model predictions by Mellon et al. [2], which identified a critical threshold north of 50° N where subsurface water ice can be stable at depths of less than 1 m. In contrast, scalloped depressions and brain terrain are more prevalent at mid-latitudes, and their distribution does not fully coincide with the modern water ice stability boundary, suggesting that these two landform types may largely record the degradation and reworking processes of ancient, buried ice under subsequent climatic fluctuations. The peak abundances and latitudinal decline of these landforms agree well with the findings by Sako et al. [50], who demonstrated a spatial association between mid-latitude periglacial landforms on Mars and simulated high annual water ice budget zones.

This zonal contrast agrees broadly with earlier interpretations that different periglacial landforms in Utopia Planitia may correspond to different expressions or stages of ice-related surface and near-subsurface processes. In particular, thermal contraction cracks are often discussed as indicators of shallow ice-cemented ground, whereas scalloped de-pressions and brain terrain are more commonly linked to degradation or modification of ice-rich materials.

From a regional perspective, the latitude determines the macro-scale background for the development of periglacial landforms, while the longitude further controls their local distribution intensity and assemblage patterns. The latitudinal variation reflects differences in the temperature field and the stability depth of shallow subsurface ice, whereas the longitudinal variation is more closely related to sediment thickness, slope conditions, and local geological context. For instance, thermal contraction cracks are most densely developed between 100° E and 110° E, which coincides with the depositional center of Utopia Planitia. This longitudinal peak is supported by SHARAD radar evidence of thick (80–170 m), high-purity (55–85% water ice) subsurface deposits in this region [11], as well as by Zhurong rover radar observations revealing layered basin-fill sequences from the Late Hesperian to Amazonian [13]. The combined effect of these factors produces the observed differentiation of periglacial landform types and densities across the Utopia Planitia. This finding suggests that periglacial landforms in the mid-latitudes of Mars can not only be used to indicate the distribution of modern near-surface water ice but also serve as important geomorphic evidence for reconstructing paleoclimate environmental evolution.

The high periglacial landform density in fine-grained sedimentary units supports the interpretation that the Vastitas Borealis Formation provides an ideal substrate for ice-wedge polygons and sublimation degradation [38,48]. The low coverage in volcanic units aligns with the expectation that dense lava inhibits periglacial modification [47]. Furthermore, the interpretation that scalloped depressions form through sublimation degradation of ice-rich layers, with thermal contraction cracks developing around them [51], supports our observation that flat-floored depressions likely represent early-stage depressions in areas with deeper ice or weaker freeze–thaw cycles. The high proportion of brain terrain in AHv suggests local remnants of ice-rich fine-grained material, consistent with the view that periglacial landforms are controlled by shallow subsurface ice distribution rather than strictly by geological unit [49]. The close spatial association between brain terrain and thermal contraction cracks observed in our study is also consistent with Levy et al. [9], who noted that brain terrain in Utopia Planitia is often co-located with polygonal patterns and latitude-dependent mantling textures. These consistencies confirm that the model predictions possess clear geological significance.

It should be noted that the distribution analysis in this study is still subject to limitations related to data sources and methodological conditions. As illustrated in Figure 10, the model exhibits omission and misclassification in low-contrast and texture-similar regions, particularly for fine-scale thermal contraction cracks and for features resembling aeolian streaks or crater rims. However, in some areas where brain terrain and thermal contraction cracks co-occur, the model’s inability to assign a strict class label may not solely reflect algorithmic failure. Instead, such mixed or ambiguous classifications may capture the physical reality that these landforms are undergoing continuous transitional degradation. This process forms geomorphological ecotones—transitional boundaries analogous to ecological ecotones—where one periglacial landform gradually merges into another. Regarding the potential influence of projection distortion, the model was trained on 512 × 512 pixel patches extracted from the Plate Carrée mosaic. Within such a local patch, the scale variation due to latitude is negligible, so the landform textures used for recognition are essentially unaffected. Furthermore, our key conclusions rely on relative density differences across latitude–longitude bins, not on absolute area measurements across latitudes. Hence, the use of a reprojected mosaic does not compromise model generalization or the observed spatial patterns. The grid statistics using cells of 10° in longitude and 5° in latitude are more suitable for revealing macro-scale distribution patterns but may exert a smoothing effect on local anomalous concentration areas, leading to the weakening of some peak characteristics. Future work should address the spectral ambiguity and low-contrast limitations by integrating multi-source data, such as combining optical CTX/HiRISE imagery with SHARAD radar sounding or high-resolution DEM topography, to reduce misclassification and omission rates in future regional-scale mapping.

Future work should further integrate multi-source remote sensing and geophysical data to address the spectral ambiguity and low-contrast limitations identified in this study. For instance, the SHARAD radar sounding data have been successfully applied to detect shallow water ice in central Utopia Planitia [52], and combining such radar evidence with optical CTX/HiRISE imagery could help validate whether regions of mixed classification (e.g., brain terrain co-occurring with thermal contraction cracks) correspond to transitional degradation zones with variable ice content. Moreover, the crustal structure of the Utopia impact basin [53] may exert a first-order control on the thickness and preservation of ice-rich sediments, thereby influencing the large-scale distribution patterns of periglacial landforms. Future studies could explore how basin-scale accommodation space affects the longitudinal clustering. The diurnal temperature cycle models developed from in-situ and satellite data [54] offer a pathway to quantitatively link the stability of shallow ground ice with the latitudinal zonation of thermal contraction cracks and flat-floored depressions, moving beyond qualitative correlations. In addition, the presence of transverse aeolian ridges in the Zhurong rover landing area [55] suggested that aeolian processes might partly contribute to the misclassification of certain textured landforms (e.g., brain terrain versus wind streaks). Integrating high-resolution topographic data (e.g., HiRISE DEM) could help disentangle periglacial from aeolian signatures. Finally, the automated crater detection approach [56] demonstrated the feasibility of machine learning-based geomorphic mapping at planetary scales. Extending such methods to periglacial landforms—by incorporating SHARAD-derived ice stability maps and thermal modeling outputs—would enable a more robust and semi-automated pipeline for future regional-to-global assessments of Martian periglacial environments, while also reducing the omission and misclassification rates.

## 5. Conclusions

This study developed the MTU-Net model for automatic segmentation and regional mapping, and identified four representative periglacial landforms on the Utopia Planitia, Mars using CTX images. Main conclusions are summarized as follows:

(1) The proposed MTU-Net, which combines multi-scale texture enhancement with a hierarchical attention strategy, performed well on the constructed dataset. Under the same evaluation protocol, the full model reached an mIoU of 89.55%, an F1-score of 94.71%, and a Kappa coefficient of 91.21%, exceeding the traditional U-Net and the two comparison models in this study.

(2) The trained model using CTX images produced regional maps of flat-floored depressions, thermal contraction cracks, scalloped depressions, and brain terrain across the study area. These maps show clear spatial heterogeneity, expressed mainly as latitudinal zoning and longitudinal clustering, based on the coverage density statistics of the four periglacial landform types. For latitudinal zoning, thermal contraction cracks and flat-floored depressions peak at 50–55° N, whereas scalloped depressions and brain terrain peak at 40–50° N. For longitudinal clustering, thermal contraction cracks are concentrated at 100–110° E, brain terrain at 110–120° E, and scalloped depressions at 90–100° E. These patterns provide a practical basis for regional geomorphological analysis in Utopia Planitia.

(3) Thermal contraction cracks and flat-floored depressions are concentrated mainly between 50° and 55° N, whereas scalloped depressions and brain terrain are more common between 40° and 50° N. This distribution is broadly consistent with earlier interpretations that different periglacial landforms in Utopia Planitia may reflect different expressions or stages of ice-related surface and near-subsurface processes.

(4) Despite uncertainties related to image resolution, local classification errors, and the spatial scale of the density statistics, the present workflow shows that CTX-based regional mapping of Martian periglacial landforms is feasible. Future work should strengthen the geomorphological interpretation by combining higher-resolution imagery, topographic information, radar observations, and formal uncertainty analysis.

## Figures and Tables

**Table 1:** Number of Dataset Categories.

Landform Type	Area of the Surveyed Terrain/km^2^	Number of Samples	Image Proportion
Flat-floored Depressions	739.78	474	29.94%
Thermal Contraction Cracks	1016.09	502	31.71%
Scalloped Depressions	411.71	347	21.92%
Brain Terrain	228.81	262	16.55%

**Table 2:** Computational cost comparison of different models.

Model	Number of Parameters/M	Training Time/h	FPS
U-Net	0.22	11.56	17.24
DeepLab	39.12	14.33	16.15
C2Fnet	34.21	8.35	31.67
MTU-Net	0.23	11.08	17.41

**Table 3:** Ablation experiment data.

Base	+A	+B	mIoU/%	F1/%	mAcc/%	Kappa/%	ACIoU/%	BCIoU/%	CCIoU/%	DCIoU/%
√			84.22	90.86	88.92	86.64	83.48	85.26	86.92	81.21
√	√		85.49	92.54	85.83	88.27	84.56	85.79	87.83	83.79
√		√	87.12	93.87	87.22	90.33	86.32	87.39	87.88	86.89
√	√	√	89.55	94.71	89.88	91.21	89.02	88.39	91.88	88.89

**Note:** Ablation experiments were configured with four settings: Base (baseline U-Net), +A (with attention mechanism), +B (with multi-scale texture module), and +Both (with both modules). ACIoU, BCIoU, CCIoU, and DCIoU denote the IoU values for flat-floored depressions, thermal contraction cracks, scalloped depressions, and brain terrain, respectively.

**Table 4:** Relative proportion of each category in different geological units.

Geological Unit	Area of Geological Unit/km^2^	Flat-Floored Depressions/%	Thermal Contraction Cracks/%	Scalloped Depressions/%	Brain Terrain/%
Amazonian and Hesperian impact (Ahi)	65,888.4014	4.98	24.41	35.66	34.95
Amazonian and Hesperian volcanic (AHv)	6456.6619	0.66	6.27	0.23	92.83
Amazonian volcanic (Av)	528,973.4817	3.35	20.79	8.95	66.91
early Amazonian basin (eAb)	736,817.1715	35.89	5.64	24.43	34.03
late Hesperian lowland (lHl)	2,220,805.9655	48.80	12.55	16.70	21.95
middle Amazonian lowland (mAl)	801,529.5857	33.59	18.51	23.58	24.33

**Table 5:** Major Spatial Distribution Characteristics of Four Types of Periglacial Landforms and Their Potential Environmental Indicative Significance.

Landform Types	Primary Enrichment Zone	Spatial Distribution Characteristics	Possible Environmental Implications
Flat-floored Depression	50–55° N, locally 110–120° E	Widest distribution, often appears as transitional type	May reflect widespread shallow periglacial modification or a transitional morphology associated with surrounding periglacial landforms
Thermal Contraction Crack	50–55° N, 100–110° E	High-latitude concentration, distinct reticulated continuous distribution	Commonly interpreted as being associated with shallow ice-cemented ground and freeze–thaw-related cracking
Scalloped Depression	40–45° N, 90–100° E	Local clustered distribution, relatively low density	Commonly interpreted as being associated with sublimation degradation of ice-rich materials
Brain Terrain	45–50° N, 110–120° E	Contiguous distribution in the east, fine texture	Indicates slow creep of ice-rich sediments and ice degradation

## Data Availability

The CTX global mosaic data used in this study are publicly available from the Murray Lab at Caltech (https://murray-lab.caltech.edu/CTX/V01/tiles/ (accessed on 18 May 2026)). The annotated dataset generated in this study is available from the corresponding author upon reasonable request. The source code for the MTU-Net model is available at https://aistudio.baidu.com/projectdetail/10150487?sUid=17544399&shared=1&ts=1774960285979 (accessed on 31 May 2026).

## Abbreviations

The following abbreviations are used in this manuscript:

CTX	Context Camera
MRO	Mars Reconnaissance Orbiter
HiRISE	High Resolution Imaging Science Experiment
MTU-Net	Multi-scale Texture Enhanced U-Net
mIoU	mean Intersection over Union
mAcc	mean Accuracy
CBAM	Convolutional Block Attention Module
EMSA	Enhanced Multi-scale Autocorrelation Texture Module
